# Development of Epstein Barr virus biosensor by mimicking its infection mechanism for early detection of multiple sclerosis

**DOI:** 10.1038/s41598-025-13030-2

**Published:** 2025-08-01

**Authors:** Ülkü Anik, Nil Su Çaylayik

**Affiliations:** 1 Chemistry Department, Faculty of Science, Mugla Sitki Kocman University, 48000 Mugla, Turkey; 2 Sensors, Biosensors and Nano-diagnostic Systems Laboratory, Research Laboratory Center, Mugla Sitki Kocman University, 48000 Mugla, Turkey

**Keywords:** Diagnosis, Sensors

## Abstract

Given the established link between Epstein-Barr virus (EBV) and multiple sclerosis, the early and asymptomatic detection of EBV has become increasingly important. In this study, we developed an impedimetric EBV biosensor designed for practical and early screening, based on mimicking the virus’s natural infection mechanism. To achieve this, the B cell surface protein CD21—serving as the host receptor—was immobilized onto a carbon screen-printed electrode. The interaction between CD21 and the EBV surface glycoprotein gp350 was monitored using electrochemical impedance spectroscopy. Following the optimization of experimental parameters, the biosensor’s analytical performance was evaluated. It exhibited a linear detection range from 5 to 100 ng/mL, with a limit of detection of 0.0074 ng/mL, a limit of quantification of 0.022 ng/mL, and a relative standard deviation of 1.34%. The optimized biosensor was then tested using 1:100 diluted saliva samples collected from healthy individuals, yielding highly promising results that support its potential for real-world applications.

## Introduction

Epstein-Barr virus (EBV), a gamma herpesvirus 4, is one of the most common human viruses in the world. EBV can be described as highly infectious, considering 90% of the world’s population is infected with EBV. Although generally harmless, EBV can remain in the human body throughout life and is the cause of infectious mononucleosis (IM) and some lymphoid and epithelial cancers including Burkitt’s lymphoma, Hodgkin’s disease, nasopharyngeal carcinoma and gastric cancer. In this concept, EBV is the first identified human oncogenic virus and defined as a Class I oncogenic virus by the World Health Organization. In addition, EBV infection is associated with a number of autoimmune diseases, including multiple sclerosis (MS) and systemic lupus erythematosus^1^.

MS can be described as heterogenous autoimmune disease. Briefly in MS, the myelin sheath of the central nervous system was attacked by immune system, particularly, autoreactive lymphocytes. As a result of this attack, many areas of myelin have been destroyed by leaving behind scar tissues that are called sclerosis. As a result of this nerve damages, electrical signals can not be transported properly to and from the brain. This situation has effects that differ from person to person since some MS patients have mild symptoms, others may have intense loss of abilities like seeing, walking and even speaking. MS is usually diagnosed by following symptoms and signs and by conducting imaging tests, and lab tests^2^. However, considering its effect in the human living quality, early and practical detection of MS is important.

Meanwhile although there are reports in the literature which suggests the relationship between EBV infection and MS, this situation could not be proven until 2022. In that study, MS diagnosed US Army personnel’s longitudinally collected saliva samples who had been followed more than 20 years and selected among 20 million personnel, were monitored for anti-EBV antibodies. As a result, it has been found out that, the personnel who was positive in terms of EBV, had a 32-fold more chance to get MS than individuals who were free of EBV^3^. For this reason, early screening of EBV could result with early diagnosis of MS.

For EBV diagnosis techniques like immunofluorescence, viral culture, enzyme-linked immunosorbent assay (ELISA), and polymerase chain reaction (PCR) have been used. EBV-specific antibodies in saliva samples are also analyzed and accepted as the gold standard for EBV detection. However, all of these diagnosis techniques suffer from serious challenges that limit their applications for EBV diagnosis. For example, ELISA contains numerous detection steps, long incubation durations and unfortunately chance of possible false positive results due to antigen–antibody cross-reactions. Although PCR shows high sensitivity, since it includes many analytical steps and can easily open to cross contamination, it lacks from practicality. For saliva antibody detection technique, the main challenge happens to be slow results which is not very preferable when rapid results are needed^4^. Considering all of these, it is obvious that, practical, rapid, accurate, sensitive and selective EBV tests that can be suitable for early screening, are still needed to be developed.

Meanwhile, it has been reported that EBV infections start at saliva and then move mostly to naïve B cells onto oropharynx and tonsils of the targeted host. During that process, since new particles are produced, it is claimed that EBV transmission can possibly occur both for memory B cells and epithelial cells as well. On the other hand, it is definite that EBV creates lifelong persistence throughout an individual’s life^1,5^. The virus’s preference for B cells plays a central role in its pathogenesis. While primary EBV infections are typically asymptomatic during childhood, delayed infection in adolescents or young adults can result in IM, which is characterized by the proliferation of EBV-infected B cells and an active T-cell response^6^. IM is linked to an increased risk of developing conditions such as Hodgkin’s lymphoma and MS^7,8^.

EBV has similar viral entry path as other enveloped viruses. The initial infection starts by binding of viral glycoproteins onto host cell’s suitable receptor. However, unlike the similar viruses, for EBV there is a variety of glycoproteins that can bind various receptors on the host cell. For example, EBV envelope glycoprotein gp350/220 interacts with host B cell CD21/CD35 and as a result, EBV is attached onto the host cell^9^. The mechanism of this attachment is given as gp350/220 binds to CD21 via its N-terminal region, spanning amino acids 1–470, which contains a glycan-free surface patch^10,11^. This predicted interaction region on gp350 exhibits a highly negative electrostatic potential, while the corresponding SCR1–SCR2 domains of CD21 present a positively charged surface. These contrasting charges suggest the likelihood of electrostatic interactions^11^. Additionally, the morphological features of the two surfaces appear to be complementary. However, direct structural data of the gp350-CD21 complex are still lacking, and the precise details of their interaction remain to be confirmed^1^.

Following this, in this study an electrochemical EBV biosensor was fabricated by mimicking above interaction for the first time. Generally, similar EBV biosensors are based on antigen–antibody interaction, which usually needs longer times for obtaining results. In this sense, with this biosensor, it is aimed to fabricate a rapid and robust system which is able to detect asymptomatic lifelong persistent EBV in B-cells.

Recently our group developed various electrochemical and colorimetric biosensors that were based on infection mechanisms of influenza, SARS-CoV-2 and Monkeypox viruses^12–14^. In this study, in order to mimic infection mechanism, CD21 was attached onto the electrode surface and impedance differences were monitored via electrochemical impedance spectroscopy (EIS) upon immobilization of gp350 onto this surface. By developing this system, it is aimed to create a screening test that can be applied to children where EBV infection shows almost no symptoms or symptoms are indistinguishable from other mild, short-lived childhood illnesses.

## Experimental

### Materials

Potassium dihydrogen phosphate (KH_2_PO_4_) and sodium hydroxide (NaOH) were bought from Merck. K_3_Fe(CN)_6_, K_4_Fe(CN)_6_, N-hydroxysuccinimide (NHS), and N-(3-((dimethylamino)propyl-)-N’-ethylcarbodiimide (EDC) were obtained from Sigma-Aldrich. Recombinant Human CD21 and recombinant EBV protein gp350 were provided from Sino Biological Inc. Carbon screen-printed electrode (cSPE) was purchased from Dropsens. All reagents and chemicals used in this study were of analytical grade and were employed as received from commercial suppliers. Additionally, all other solutions were prepared using ultrapure water obtained from the Bluaqua Kapelle series ultrapure water system.

### Instrumentation

A commercially available cSPE was chosen as the transducer for developed EBV immunosensor. This cSPE featured a carbon working electrode with a 4 mm diameter, along with a carbon auxiliary electrode and a silver reference electrode, all integrated into a single platform. EIS measurements were employed, using a μ-AUTOLAB potentiostat, which was equipped with NOVA 1.10 software and an FRA-2 module. Atomic force microscopy (AFM) analyses were conducted in tapping mode, utilizing resonant frequencies between 15 and 29 kHz. All images were obtained in air under ambient conditions. On the other hand, the samples were coated with a 9 nm gold/palladium (Au/Pd) layer via Leica EM ACE600 sample coater before performing the scanning electron microscopy (SEM) measurements. Imaging was performed at a chamber pressure of 1.00–3 Pa, achieving a resolution of 1 nm at 1 kV. Infrared spectra were recorded using the attenuated total reflectance mode on a Nicolet iS50 Fourier Transform Infrared (FTIR) spectrometer (Thermo Scientific Inc.), covering the wavenumber range of 1–4000 cm^−1^. Lastly, incubation processes were conducted using a BIOSAN ES-20 environmental shaker incubator.

### Procedure

**Fig. 1:**
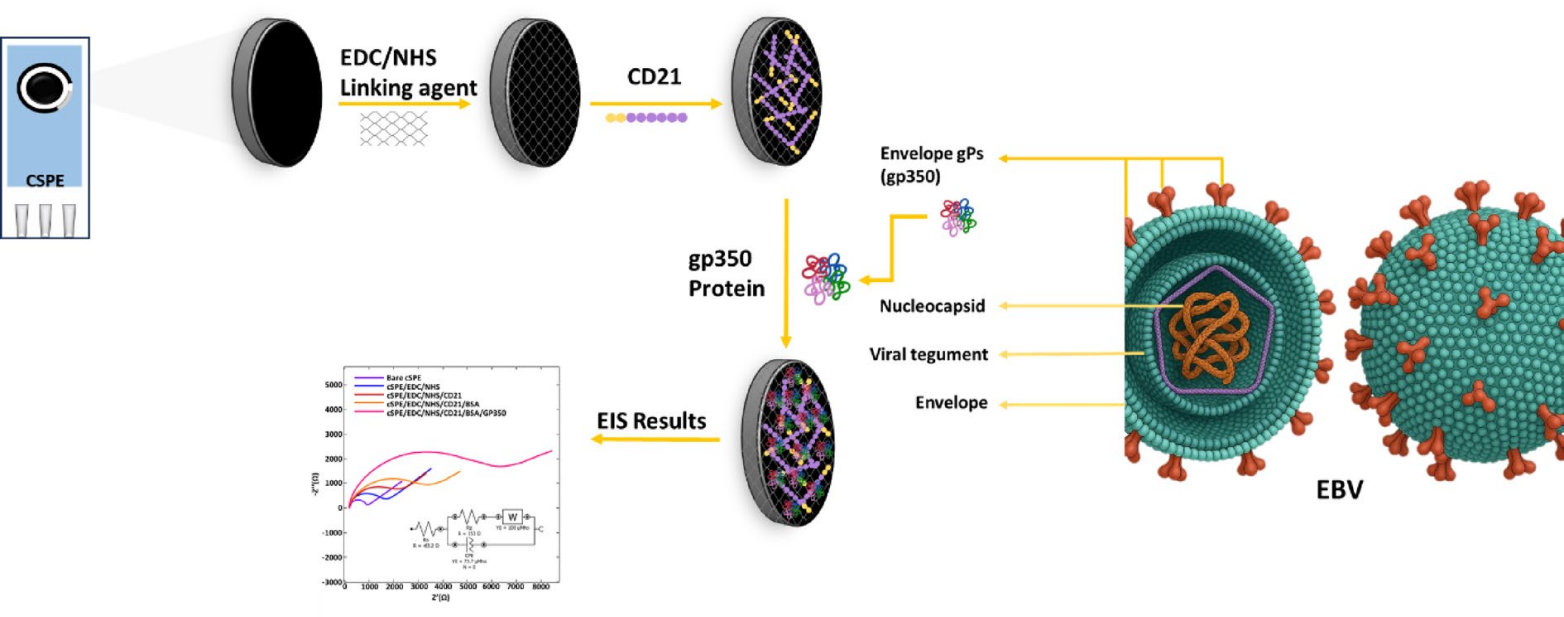
Schematic representation of the fabrication of EBV biosensor.

10 μL of 100 mM EDC and 10 μL of 150 mM NHS were placed onto cSPE surface as the first step of EBV biosensor fabrication procedure. Then this surface was dried by applying 25 °C for 15 min. Following that, 10 μL of CD21 (20 ng/mL^−1^ in pH: 7.0 phosphate buffer solution (PBS) was attached onto previous layer of electrode surface for 30 min at 25 °C. After that, %0.1 BSA was prepared and dipped onto the cSPE surface and dehydrated at 25 °C for 15 min. Eventually, proper amount of gp350 protein was immobilized onto CD21-BSA@cSPE and incubated at 37 °C for 30 min (Fig. 1). After each preparation step and before applying EIS technique, the surface was washed with 200 μL of 0.1 M PBS. Meanwhile for conducting EIS, 0.1 Hz and 100 kHz range was selected as the working frequency where applied potential was 0.1 V. The resistance difference that belonged to CD21-BSA@cSPE and gp350-CD21@cSPE was obtained in the presence of 50 μL of 5 mM [Fe(CN)_6_]^3−/4−^ redox probe couple, while 1.5 mL of PBS (pH: 7.0) was utilized for rinsing process among all fabrication steps. The main aim of the measurement process was based on monitoring the above mentioned resistance difference. Meanwhile all the experiments were repeated for three times and obtained results were presented with error bars.

### Selectivity study

The A27L antigen of the Vaccinia virus, the H3N2 antigen of the Influenza A virus, and the KMP11 protein found in Leishmania are biological agents capable of interacting with CD21. Therefore, these three agents were selected for interference studies and tested at a 1:1 ratio in the presence of 50 ng mL^−1^ gp350.

### Real sample application

Sample analyses were carried out by introducing specific concentrations of gp350 into saliva samples diluted at a 1:100 ratio, obtained from one healthy individual. Saliva samples were collected after cleansing the teeth and mouth, after any meal during the day. Prior to saliva collection, chewing of pure/sugar-free gum for 5–10 min within a 2-h period was done. gp350 was added at concentrations of 5, 10, 25, and 50 ng mL^−1^, for three collected saliva samples and measurements were conducted in triplicate. The results obtained from these replicates were compiled and represented as a calibration curve. We confirmed that all experimental protocols were approved by Muğla Sitki Koçman University Medical and Health Sciences Ethics Committee with protocol number of 240205 and decision number of 160 and the need for informed consent was also waived by Muğla Sitki Koçman University Medical and Health Sciences Ethics Committee.We also would like to mention that all methods were performed in accordance with the relevant guidelines and regulations. Apart from that, we are confirming that informed consent was obtained from all subjects and/or their legal guardian(s).

### Storage stability

The stability of the developed EBV biosensors was checked by following the signal of a single electrode. For this purpose, the same biosensor performance was observed at specific time intervals after 50 ng/mL^-1^ gp350 was immobilized onto the electrode surface. These time intervals included per day for five days, per week that lasted for one month totally. Between each measurement, the electrode surface was rinsed with PBS buffer and was kept at 4 °C.

## Results and discussion

### Characterization of fabricated EBV biosensor

**Fig. 2:**
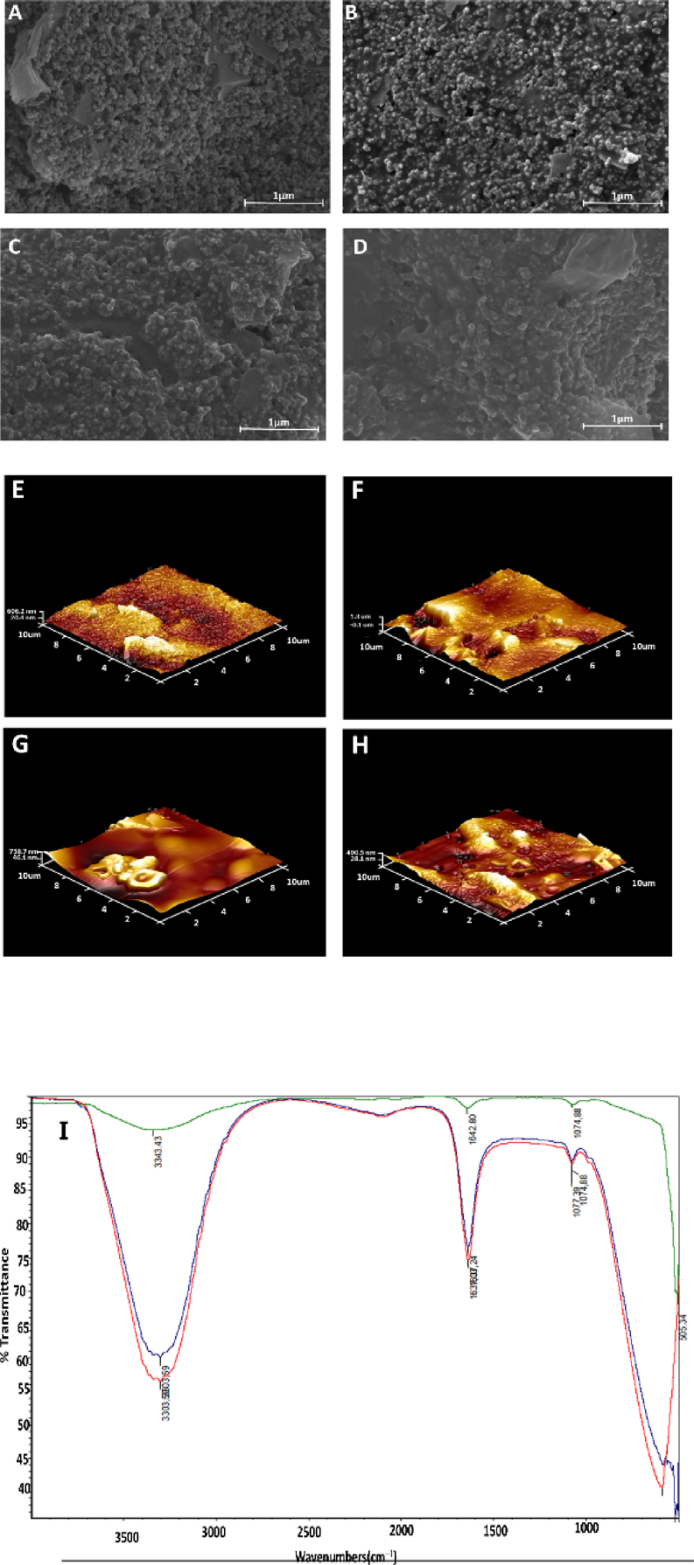
SEM (**A**–**D**), AFM (**E**–**H**) and FTIR (I) images of bare cSPE, CD21@cSPE, BSA@CD21@cSPE andgp350@BSA@CD21@cSPE respectively.

For the step-by-step characterization of the EBV biosensor, apart from electrochemical characterization, SEM, AFM and FTIR analyses were conducted and are demonstrated in Fig. 2.

In Fig. 2A– D the SEM images of bare cSPE, CD21@cSPE, BSA@CD21@cSPE and gp350@BSA@CD21@cSPE are shown respectively. It is clear from the images that upon addition of CD21, bright dots were formed on the electrode surface compared to plain cSPE (Fig. 2A, B). Meanwhile, the introduction of BSA on the same surface covered these bright dots, forming a kind of layer (Fig. 2C). The attachment of gp350 is observed in Fig. 2D by an increment of coverage, hence layer formation compared to Fig. 2C.

On the other hand, Fig. 2E –H demonstrates the 3D AFM images of bare cSPE, CD21@cSPE, BSA@CD21@cSPE and gp350@BSA@CD21@cSPE respectively. As can be seen from Fig. 2E, bare cSPE has a grainy surface. Upon binding of CD21, a coverage was seen on the surface (Fig. 2F). When BSA was added onto CD21, the level of coverage increased by forming a smoother layer together with heterogeneous accumulations on the side (Fig. 2G). Finally, the immobilization of gp350 was observed by forming more heterogeneous craters on the electrode surface (Fig. 2H).

In conclusion, the observed variations in surface morphology and roughness confirm the sequential modification steps in biosensor construction, supporting the effective layer-by-layer assembly for sensitive and specific EBV detection.

The FTIR spectra presented in Fig. 2I illustrate comparative analyses about the binding of three molecules: the red trace corresponds to the CD21 antibody alone, the blue trace represents the CD21-BSA complex, and the green trace denotes the ternary system involving CD21, BSA, and the gp350 antigen.

The red spectrum, representing the free CD21 antibody, exhibits characteristic amide I and amide II bands at − 1650 cm^−1^ and − 1540 cm^−1^, respectively, which are attributed to C=O stretching and N–H bending vibrations of the peptide backbone. A broad absorption around 3300 cm^−1^ corresponds to N–H and/or O–H stretching, indicative of hydrogen bonding and the presence of secondary structure elements^15^.

Upon complexation with BSA (blue spectrum), subtle shifts and intensity changes are observed in the amide I and II regions. This suggests the occurrence of intermolecular interactions, likely through hydrogen bonding or electrostatic forces, between CD21 and BSA. The change of bands in the 1200–1000 cm^−1^ region further implies alterations in the microenvironment around functional groups, possibly due to changes in the tertiary structure or new bonding motifs forming between the two proteins^15^.

The green spectrum, representing the CD21-BSA-gp350 ternary complex, shows further modifications compared to the binary system. A notable broadening and shift of the O–H/N–H stretching region (− 3300 cm^−1^) and changes in the intensity of the amide I and II bands indicate additional conformational rearrangements and new interaction sites, likely due to the binding of the gp350 antigen. These spectral changes support the hypothesis of a multivalent interaction, potentially involving cooperative binding or conformational adjustments in response to gp350 association^15^.

**Fig. 3:**
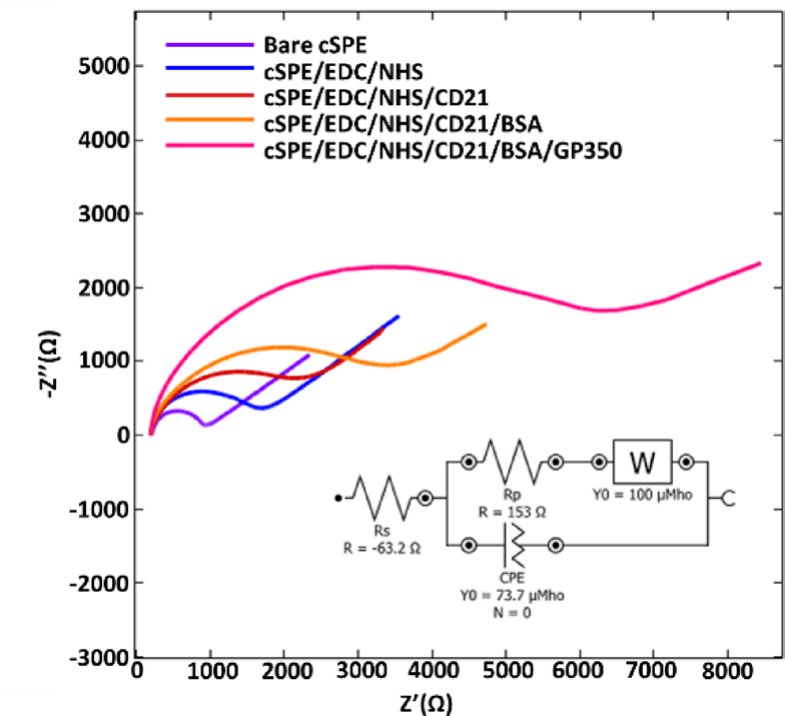
The electrochemical characterization of impedimetric EBV biosensor. surface. The system is modeled with an equivalent circuit comprising RSR (solution resistance), Rp (polarization resistance) Rs (solution resistance) RCT (interface resistance), CPE (constant phase element), and W (Warburg impedance), ΔR: Resistance difference, R^2^ (chi-squared).

Apart from structural characterization studies, electrochemical characterization of developed EBV biosensor had also been done (Fig. 3). From the figure, it is obvious that plain cSPE has the smallest radius in terms of the Nyquist curve since no layer has been formed on its surface. Upon immobilization of EDC-NHS onto the cSPE surface, due to a new layer formation on the electrode surface, an increase in the Nyquist curve’s radius was obtained. Here, it is believed that EDC attaches onto the electrode surface with the help of functional groups like COOH or OH that are found on the surface, and NHS stabilizes the intermediate formed from the reaction of EDC with the suitable functional groups^16^.

In the third step of EBV biosensor fabrication, a bigger radius was observed as shown in Fig. 3C, based on the immobilization of CD21 onto the EDC-NHS layer.

Each layer formation increases the radius of the Nyquist diagrams because of the increment in the resistance on the electrode surface, which leads to partial prevention of electron transfer from and to the electrode surface. This situation continues as BSA was attached onto the CD21-included transducer surface. As the last step of the EBV infection mechanism, gp350 was attached onto the surface, which results in the expected increment on the curves, demonstrating the specific interaction between CD21 and gp350.

Moreover, the system is also modeled with an equivalent circuit as presented inset of Nyquist diagrams. In equvalent circuit, the denotations and their values are as followed: Rs (solution resistance) 184 Ω, Rp (polarization resistance) 1.88 kΩ, RCT (interface resistance), CPE (constant phase element) 2.16 μMho s^N^ (N = 0.988), and W (Warburg impedance) 100 μMho s^1/2^, ΔR: Resistance difference, R^2^ (chi-squared) 0.0023.

Meanwhile double layer capaticance (Cdl) value was calculated based on the below Eq. (1) and found as 3.93, 1.06, 0.57, 0.26, 0.074 µF for each biosensor fabrication layer respectively.

$$\left( {\omega = {2}\pi {\text{f}} = {1}/{\text{R}}_{{{\text{ct}}}} {\text{C}}_{{{\text{dl}}}} } \right)$$

Where ω is the angular frequency; f is the frequency; Rct is the difference between the endpoint and the starting point of the semicircle in the Nyquist plot and Cdl is double layer capaticance^17^.

The Cdl value for biosensor fabrication tends to decrease upon modification of each layer. This reduction in Cdl suggests either an increase in the thickness of the layer adhering to the electrode or a decrease in the effective electrode area in contact with the electrolyte^18^.

In conclusion, the electrochemical characterization studies also confirm the successful fabrication of EBV biosensor.

### Working conditions optimization studies

After selecting the CD21 concentration based on the literature ^12^, further optimizations were carried out to improve the accuracy and overall performance of the developed biosensor. Specifically, the incubation temperature and time for both CD21 and gp350 were optimized.

For these optimization studies, 50 μL of a 5 mM [Fe(CN)₆]^3−^/^4−^ redox probe was used, and EIS was performed over a frequency range of 0.1 Hz to 100 kHz at an applied potential of 0.1 V. To ensure physiological relevance, all experiments were conducted at pH 7.0, which corresponds to the typical pH of human biological fluids.

### Incubation temperature optimization studies

**Fig. 4:**
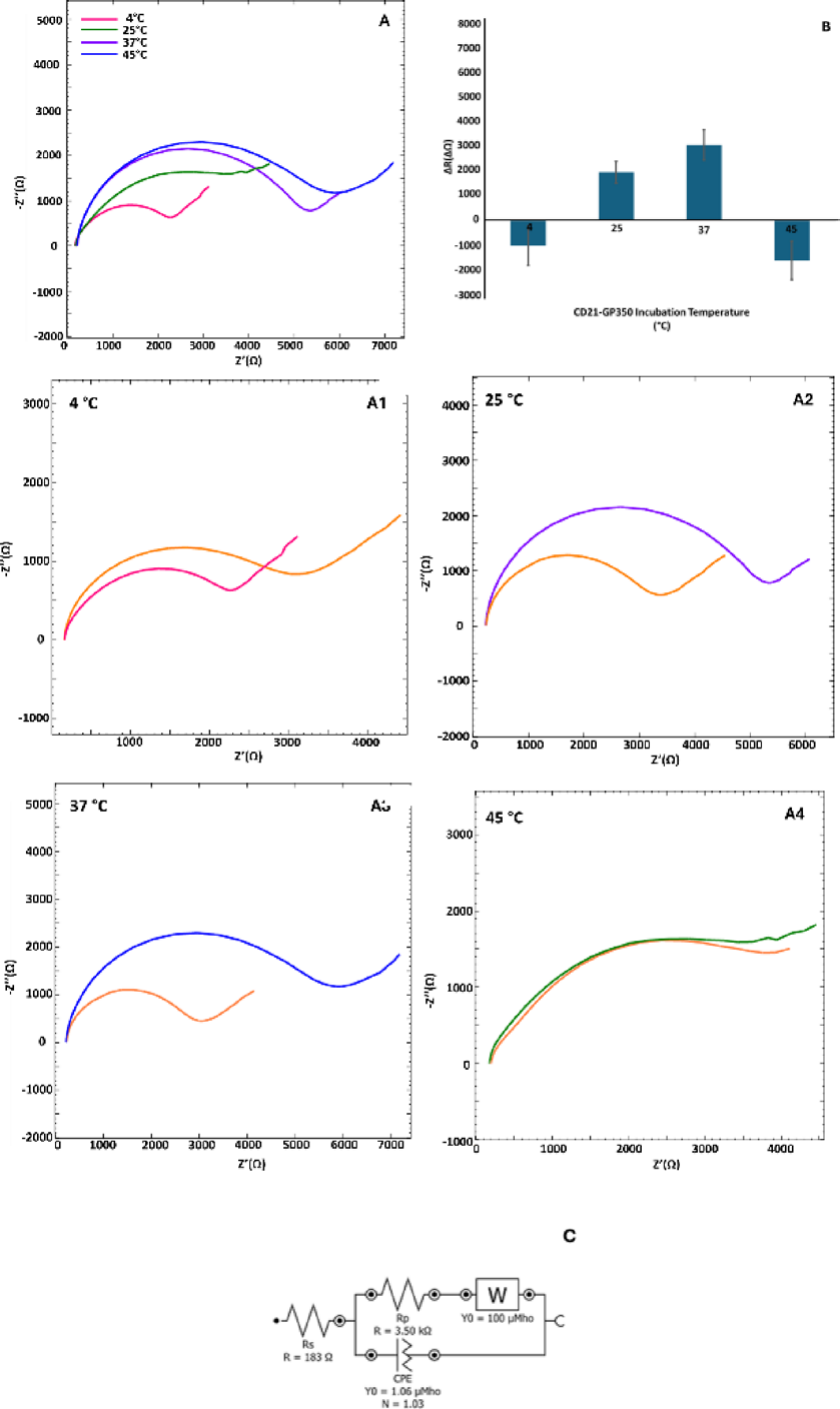
Nyquist diagrams (**A**) and impedance difference excel column charts (**B**) of CD21 and gp350 incubation temperature optimization studies for 4, 25, 37, and 45 °C (n = 3). (**C**) EIS diagrams for CD21 and gp350 interactions under conditions A1–A4, revealing differences in charge transfer resistance (RCT) and diffusion behavior (W). At the optimal 37 °C incubation, the system is modeled with an equivalent circuit. The denotations of this circuit are as Fig. 3.

As it is well known, temperature is an important parameter that highly impacts the behavior of proteins. Proteins can act differently under high and low temperatures^19, 20^. So, following this fact, a series of incubation temperatures (4, 25, 37, and 45 °C) were researched for effective interaction of CD21 and gp350. Based on the obtained resistance differences from EIS measurements, the best incubation temperature was determined as 37 °C (Fig. 4). It has been known that the capability of a protein to change its conformation towards the temperature change is especially important in regions where binding and catalysis occur.^19^. Also, it has been reported that temperature related misshape of these regions might affect the functioning negatively, which might be the case that happened here. For this reason, 37 °C was chosen as the optimum incubation temperature and used for further studies.

### Optimization of CD21-gp350 incubation time

**Fig. 5:**
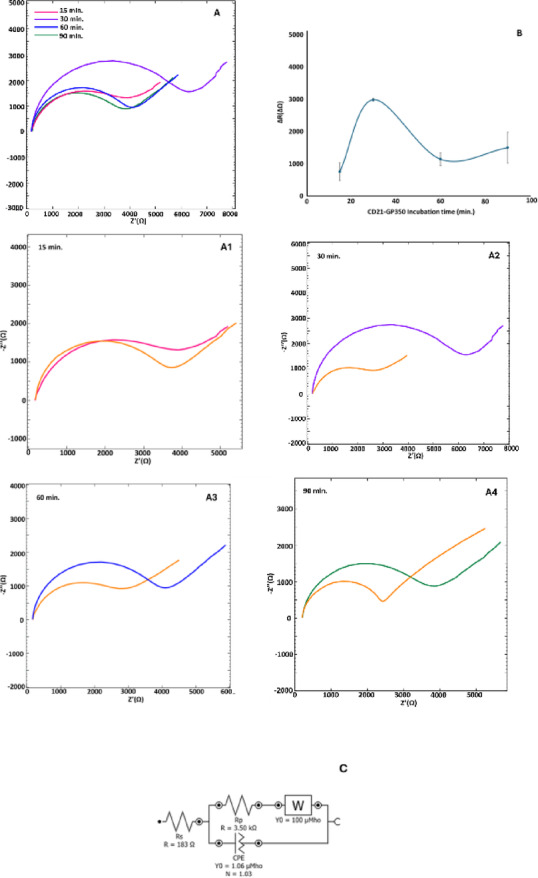
Nyquist diagrams (**A**) and regarding resistance difference excel plot (**B**) of CD21 and gp350 incubation time optimization studies for 15,30, 60, and 90 min (n = 3). Close up looks of impedance differences regarding CD21 and gp350 interaction (A1–A4) and equivalent circuit (**C**). The parameters of equivalent circuit are as in Fig. 3.

Optimizing incubation time is a crucial step in achieving a sensitive and effective immunosensor. To determine the optimal duration, the developed EBV immunosensor was tested with incubation times of 15, 30, 60, and 90 min. As shown in Fig. 5, the highest resistance change was observed at 30 min. Given the balance between performance and practicality, 30 min was selected as the optimal incubation time and was used in all subsequent experiments.

### Analytical characteristics

**Fig. 6:**
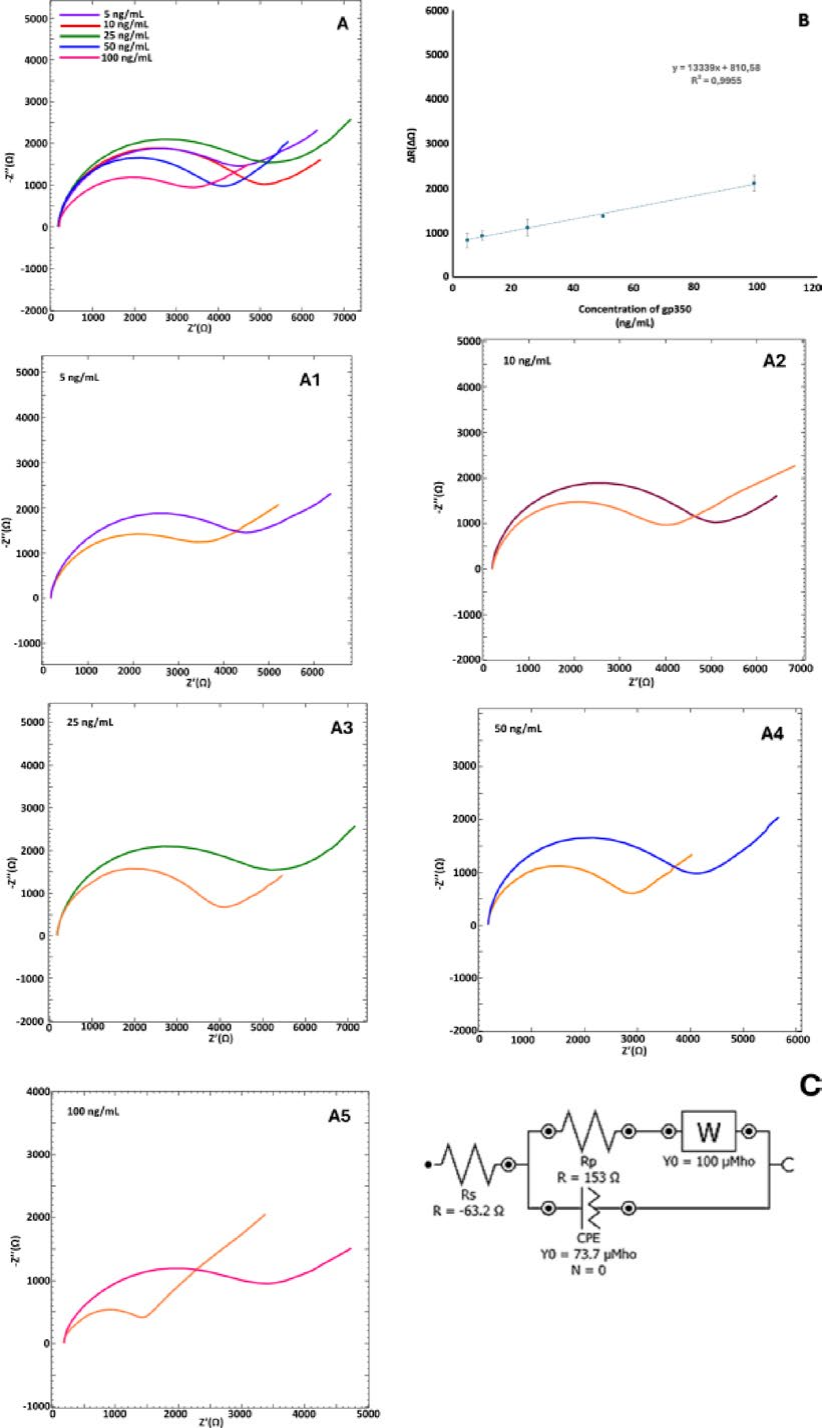
(**A**) EIS diagrams and (**B**) calibration curve regarding to gp350 concentrations of 5.0, 10.0, 25.0, 50.0, 100.0 ng mL^−1^. (A1–A5) Demonstration of resistance difference about concentrations of 5.0–100.0 ng mL^−1^ gp350 (n = 3) and (**C**) equivalent circuit; parameters are as in Fig. 3.

The analytical performance of the developed EBV immunosensor was evaluated under optimized conditions, as shown in Fig. 6, with the equivalent circuit model presented in Fig. 6C. As illustrated in Fig. 6B, a linear response was observed over the concentration range of 5 to 100 ng mL^−1^, with the calibration equation y = 13339x + 810.58 and a correlation coefficient of R^2^ = 0.9955.

The limit of detection (LOD) and limit of quantification (LOQ)—key indicators of the sensor’s sensitivity—were calculated using the standard formulas 3.3 σ/m and 10 σ/m, respectively. The LOD was determined to be 0.007 ng mL^−1^, and the LOQ was 0.022 ng mL^−1^.

**Fig. 7:**
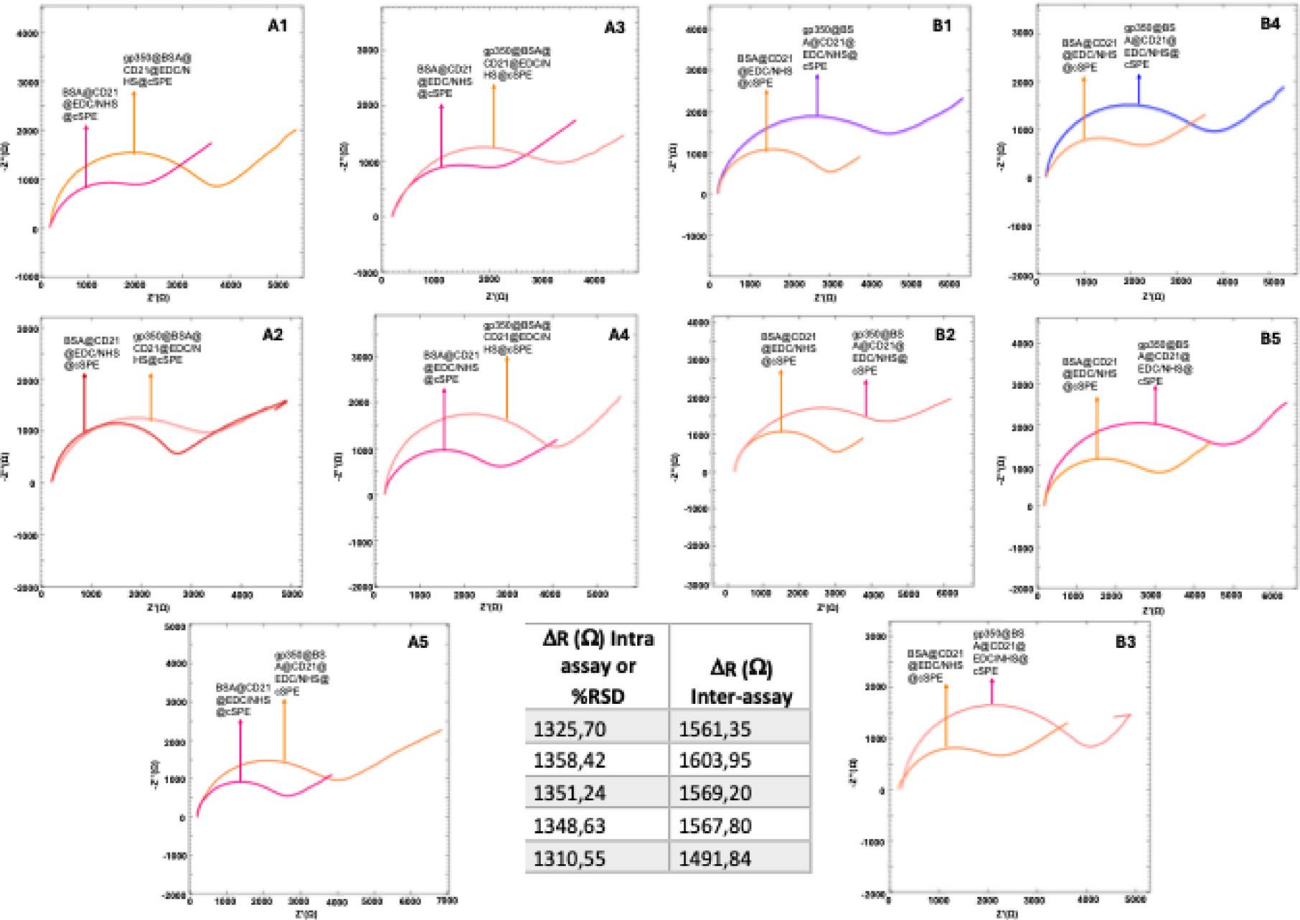
(A1–A5) Nyquist diagrams of intra assay variable and %RSD values; (B1–B5) inter assay variables. Inset table of Δ_Rct_ values for intra and inter assay studies were presented (n = 5).

Repeatability was assessed by calculating the relative standard deviation (RSD) for measurements at 50 ng mL^−1^ gp350, yielding an RSD of 1.34% (n = 5) (Fig. 7). Additionally, inter- and intra-assay variability were evaluated at the same concentration (n = 5) by using the data as shown in Fig. 7. The intra assay RSD matched the overall repeatability value of 1.34%, while the inter-assay RSD was calculated as 2.35% .

Table 1 offers a comparative analysis of the analytical performance of the developed EBV immunosensor against previously reported electrochemical EBV biosensors. In light of the obtained analytical parameters, it can be concluded that the performance of the developed immunosensor falls within acceptable limits.

**Table 1:** Comparative analysis of the analytical performance of the developed impedimetric EBV immunosensor with previously reported electrochemical EBV biosensors.

Electrode/sensor type	Method	Linear range (ng/mL)	LOD (ng/mL)	References
Polymer-modified (P4ATP)/DNA genosensor	DPV	1.2–240	5.2	^21^
AuNPs/HDT/SPA/anti-EBV/BSA/SPE	EIS	5–100	0.1	^22^
PdPtNPs/PANI/anti-LMP1/GCE	DPV	10–40	0.00062	^23^
PEI–CNTs/anti-EBNA1/SPCE	DPV	10–500	0.25	^24^
rGO–CS–AuNPs/anti-EBV/GCE	EIS	10–50	0.1	^25^
EDC-NHS/CD21/BSA/anti-EBV/ cSPE	EIS	5–100	0.007	Present work

AuNPs, Gold nanoparticles; HDT, Hexanedithiol; SPA, Staphylococcal protein A; PdPtNPs, Palladium-platinum nanoparticles; PANI, Polyaniline; CNTs, Carbon nanotubes; PEI, Polyethyleneimine; rGO, Reduced graphene oxide; CS, Chitosan; LMP1, Latent membrane protein 1; EBNA1, Epstein–barr nuclear antigen 1; GCE, Glassy carbon electrode; DPV, Differential pulse voltammetry.

Although disposable screen-printed electrodes were used to conduct this study, the storage stability of the electrodes was also investigated. Following the procedure described in the experimental section, the stability of the electrode was found to be 100% after two weeks. After one month and eight measurements, the same electrode showed a stability of 103%.

### Interference studies

**Fig. 8:**
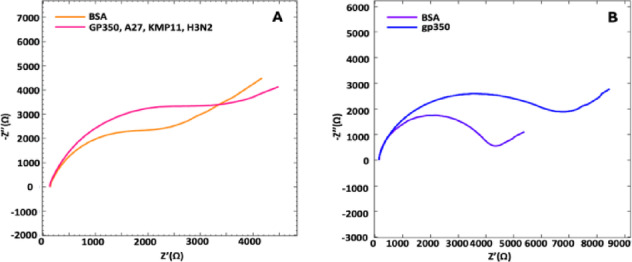
Nyquist diagrams demonstrating the resistance difference of 1:1 ratio of (**A**) different interference agents and (**B**) gp350 for interference studies (n = 3).

Interference studies were carried out under optimized conditions using a gp350 concentration of 50 ng mL^−1^, with potential interferents introduced at an equimolar ratio (1:1) relative to gp350. The selected interferents included the A27L antigen from the Vaccinia virus, the H3N2 antigen from Influenza A, and the KMP11 protein from Leishmania. EIS measurements of these 1:1 mixtures showed a recovery rate of 95.38% (n = 3). These results demonstrate the reliability and specificity of the developed immunosensor, even in the presence of these potentially interfering agents (Fig. 8).

### Sample application

**Fig. 9:**
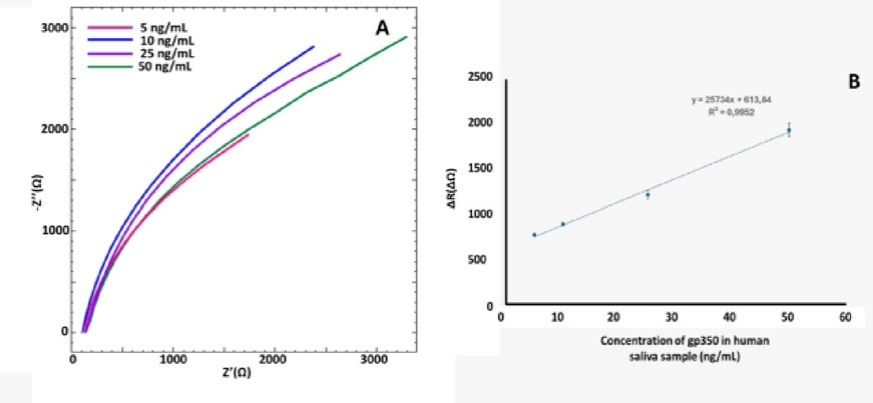
EIS diagrams (**A**) and excel plot (**B**) about 5, 10, 25, and 50 ng mL^ −1^ gp350 included 1:100 diluted saliva (n = 3 for each concentration).

For the sample analysis, properly diluted saliva samples from healthy individuals were used. To perform the study, gp350 solutions at concentrations of 5, 10, 25, and 50 ng mL^−1^ were prepared in saliva diluted 1:100. The impact of the saliva matrix on the immunosensor’s performance was evaluated using EIS, as shown in Fig. 9. A clear correlation was observed between increasing gp350 concentrations and corresponding resistance values. Table 2 presents the recovery rates and %RSD values for the sample analysis. As indicated, the developed EBV immunosensor showed no significant matrix effect from saliva at the 1:100 dilution level.

**Table 2:** Results of standard addition of gp350 to saliva samples.

Concentration (ng/mL)	RSD (%)	Recovery (%)
5	0.62	99.24
10	2.29	97.44
25	3.70	95.00
50	3.96	98.99

## Conclusion

The strong link between EBV and various cancers and autoimmune diseases, including MS, underscores the importance of early detection. In this study, we developed a practical and robust electrochemical immunosensor targeting EBV, designed around the virus’s infection mechanism. Based on our findings, we believe the sensor holds significant potential to be adapted into a diagnostic tool for detecting EBV and, potentially, for early MS diagnosis in the future.

While a 1:100 saliva dilution may not be ideal for Point-of-Care applications, it was used in this study to evaluate the biosensor’s performance in real sample conditions. For this purpose, a limited number of saliva samples were tested at four gp350 concentration levels. Moving forward, if this immunosensor is further developed into a diagnostic test for EBV or MS, a broader and more diverse set of saliva samples can be analyzed, and the dilution factor can likely be optimized based on future results.

Overall, our findings demonstrate that the interaction between CD21 and gp350 is effectively captured by the biosensor, enabling the potential early detection of asymptomatic EBV infections, particularly in younger populations. We believe that early intervention in EBV-related diseases—including MS—could significantly improve long-term health outcomes and overall quality of life.

## Data Availability

The datasets used and/or analysed during the current study available from the corresponding author on reasonable request.
